# Effects of Temperature and Salinity on Growth, Metabolism and Digestive Enzymes Synthesis of *Goniopora columna*

**DOI:** 10.3390/biology11030436

**Published:** 2022-03-11

**Authors:** De-Sing Ding, Anil Kumar Patel, Reeta Rani Singhania, Chiu-Wen Chen, Cheng-Di Dong

**Affiliations:** 1 Ph.D. Program of Aquatic Science and Technology in Industry, College of Hydrosphere Science, National Kaohsiung University of Science and Technology, Kaohsiung City 81157, Taiwan; 1041750102@nkust.edu.tw; 2 Department and Graduate Institute of Aquaculture, National Kaohsiung University of Science and Technology, Kaohsiung City 81157, Taiwan; 3 Department of Marine Environmental Engineering, National Kaohsiung University of Science and Technology, Kaohsiung City 81157, Taiwan; anilkpatel22@gmail.com (A.K.P.); reetasinghania@gmail.com (R.R.S.); cwchen@nkust.edu.tw (C.-W.C.); 4 Sustainable Environment Research Center, National Kaohsiung University of Science and Technology, Kaohsiung City 81157, Taiwan

**Keywords:** adaptability, metabolic, digestive enzymes, feeding time, body composition

## Abstract

**Simple Summary:**

Coral reefs are important habitats for marine life and have high commercial uses, which can be used in biomedicine, aquariums and tourism promotion. In recent years, climate change has posed a serious threat to coral survival. Therefore, it is very important to explore the effects of temperature and salinity changes on coral physiology. In addition to understanding the threat of climate change to corals, it can also be used as an important environmental indicator for coral large-scale aquaculture. This study aims to investigate the effects of different temperatures and salinity on the body composition, digestive enzymes and metabolism of *G. columna*, to explore the physiological and metabolic threats caused by climate change to *G. columna*, and to understand the most suitable temperature and salinity for *G. columna* growth.

**Abstract:**

Climate change is causing dramatic changes in global ocean temperature and salinity, threatening coral survival. Coral growth and metabolism are greatly affected by the temperature, salinity and feeding time of the environment. In order to explore the threats to coral survival caused by climate change, this study will investigate the changes in body composition, digestive enzymes and metabolism of *G. columna* at different temperatures and salinities. A maximum *G. columna* growth rate was observed at 25 °C and 30–35 psu salinity. The *G. columna* could survive in a wide salinity range of 25–40 psu. However, the maximum number and weight of *G. columna* polyps was determined at 30–35 psu. Furthermore, 30–35 psu salinity at 25 °C led to the best *G. columna* growth and survival, mainly because of their enhanced nutrient absorption rate, polyp expansion rate, metabolic rate and adaptability. Comparing various salinity-temperature treatment groups, all obtained values for growth, behavior and metabolism were significantly higher (*p* < 0.05) for 30 psu at 25 °C than other treatment groups resulting in maximum *G. columna* yield. In addition, the optimal timing of *G. columna* feeding was assessed by studying changes in body composition and digestive enzymes within 24 h of feeding. The results showed that *G. columna* has higher protein and protease activity between 6:00 a.m. to 12:00 noon. Therefore, at 25 °C, 30–35 psu and feeding will enhance *G. columna* growth and survival.

## 1. Introduction

Salinity and temperature are two major abiotic factors that directly affect the survival and growth of marine life [1,2]. The drastic change of salinity affects the osmotic pressure regulation and physiology of coral, resulting in the death of coral [3]. In recent years, changes in seawater temperature and salinity caused by climate change and the greenhouse effect directly affect the growth and survival of corals [2]. At present, there is no in-depth study on whether the changes in seawater temperature and salinity caused by climate change will affect the digestive enzymes and body composition of corals, which may indirectly affect reproduction, feeding, hatching and egg development. Therefore, this study will preliminarily explore whether changes in temperature and salinity will affect the digestive enzymes and physiological metabolism of corals.

Coral reefs are considered the most biodiverse ecosystems in the world, and are economically important for marine fisheries and ecotourism [4]. In recent decades, due to human over-exploitation of marine resources, pollution, global warming, imbalance of the marine ecosystem among other factors, coral reefs appear to be in crisis. Eleven percent of the world’s coral reefs are now destroyed, 16% are no longer ecologically functional and 60% are under serious threat [5]. Previous studies have pointed out that changes in salinity and temperature caused by climate change will lead to *Acropora millepora* bleaching and death, as well as preventing eggs from being fertilized smoothly [5]. The unfavorable temperature and salinity will cause the embryonic development rate of *Platygyra acuta* to decrease by 80%, which will threaten the survival of *P. acuta* [6].

In commercial applications, coral has high commercial value, ornamental and medicinal value, and can be used for research and development of antibacterial, anti-inflammatory and other drugs [4,7]. Therefore, large-scale coral aquaculture is of great help to the sustainable development of coral reefs. In some sea areas, typhoons, rainstorms and climate change have caused dramatic changes in temperature and salinity. If large-scale coral aquaculture in the sea may cause coral stress, it is necessary to rely on indoor large-scale aquaculture. Therefore, large-scale indoor coral aquaculture will be of great importance to the sustainable development of coral reefs. However, we must first understand the threats posed by temperature and salinity to corals before we can further explore the most suitable temperature and salinity for corals to survive. Previous studies have shown that low salinity and high temperature can cause a stress response in corals, resulting in decreased photosynthetic efficiency, inability to provide essential nutrients through zooxanthellae, affecting survival and growth [8,9]. Previous studies have focused on coral stress responses or physiological changes induced by changes in temperature and salinity [9]. No research has investigated whether temperature and salinity can cause changes in coral digestive enzymes, which in turn affect coral physiological metabolism. Therefore, in addition to the preliminary study on the effects of climate change on physiological metabolism and digestive enzymes of corals, this study can also find out the most suitable temperature and salinity for growth, which can be applied to the application of large-scale coral aquaculture.

In the ocean, zooxanthella often get detached from corals due to thermal stress caused by high temperature, leading to coral bleaching. A coral bleaching event occurs when reactive oxygen species rise due to environmental stress, which leads to coral death [10]. A global threat to reef ecosystem balance is coral bleaching, in which endosymbionts are expelled from coral (host) tissue as a result of heat or sometimes light stress [10]. When the temperature was further increased up to ~30–32 °C, it resulted in coral stress and bleaching [11]. Some other studies also covered how water temperature can affect coral metabolism [12]. Corals exposed to high temperature (30 °C) and low salinity (20 psu) pose stress response, resulting in the bleaching and even death of some corals [13]. Importantly, water temperature affects oxygen consumption, growth, survival and metabolism. When the temperature is low, the metabolic rate decreases the energy utilized for maintenance, which affects the efficiency of food digestion and absorption. On the other hand, when the temperature rises, the metabolism rate increases [14,15].

Corals are narrow-salt organisms with limited ability to regulate osmotic pressure. Changes in salinity and temperature caused by climate change may affect the physiological metabolism of corals [16,17,18]. *Goniopora columna* are mainly composed of tissues and skeleton, the outermost ectoderm contains nematocyst, the middle layer is mesoglea and the innermost endoderm contains zooxanthellae and mesenteries [18]. The tissue structure is quite simple, and it does not have the ability to regulate cell osmotic pressure like vertebrates. Short-term changes in salinity cause dramatic changes in cell respiration and photosynthesis of symbiotic algae [9]. Low salinity environments cause coral bleaching, i.e., a sublethal response of corals involving loss of zooxanthellae [19]. Therefore, it is worth studying whether climate change will affect the physiological metabolism of corals and cause coral growth, reproduction and death [8,20].

Previous studies by Lasker [21] and Levy [22] reported that corals have different adaptations to the environment. Adaptability can be assessed by the stretching or contracting behavior of polyps. Therefore, the adaptability of corals for temperature and salinity was evaluated and compared in this study with previously published work. The adaptability of temperature and salinity can explore the most suitable temperature and salinity conditions for columnar *G. columna* during aquaculture or artificial division and reproduction, to accelerate tissue repair and reduce the risk of death.

Biological metabolism includes both material metabolisms and energy transfer. In this study, the energy transfer caused by both material metabolisms of *G. columna* was investigated to evaluate energy generating metabolic indicators. Therefore, the animal’s oxygen consumption can be used as a good indicator of the animal’s energy utilization and physiological condition [23]. Coral cells are susceptible to oxidative damage, which changes their O_2_ metabolism and causes them to bleach, pale and die [13]. Currently, there is no research on how coral feeding, temperature and salinity changes affect oxygen metabolism. Only a few previous studies have examined coral oxygen consumption. Salinity and temperature have a major impact on physical activity and growth, as well as oxygen requirement for these functions [20]. Ammonia makes up 80% of the excretion of marine organisms, followed by amino acids and urea [24]. According to research, ammonia concentration at 0.001 mmol/L can affect chlorophyll loss of coral, but different coral species have different tolerance levels for ammonia nitrogen [25]. Therefore, attention must be paid to changes in ammonia nitrogen during coral aquaculture. At present, there are no research reports on the ammonia nitrogen metabolism of *G. columna*. This study was a preliminary investigation into whether temperature and salinity could affect the physiological metabolism and digestion and absorption of *G. columna*.

According to a study by Corner and Cowey [26], the ratio of O and N (O/N) is used as an indicator of energy metabolism, and based on the O/N values, the energy metabolism can be determined as to whether it is supported by protein or fat and the carbohydrate metabolism. When the O/N ratio is low, it means that energy metabolism is biased toward protein metabolism; when the O/N ratio is high, it is biased toward fat or carbohydrate metabolisms. In addition, when the O/N ratio is below 24, it means that its metabolism is a protein-dominated metabolism, and its energy metabolism is mainly from protein sources, and more protein is needed to prevent malnutrition or slow growth. According to the state, if the O/N ratio > 24, it belongs to the lipid-dominated metabolism type, and the energy source is mainly lipid dependent [26]. Once the metabolism type is determined, one can design the sea animals diet based on that, to obtain higher metabolic rate and associated growth. This O/N ratio can be used usually to infer type of metabolism under given environmental conditions. It is well known that both oxygen consumption and ammonia excretion of aquatic organisms are significantly affected by external environmental factors such as temperature, salinity, and water pollutants. Therefore, recent interest in investigating corals exposed to different temperature and salinity environments could reflect the definite correlation of nitrogen excretion and energy metabolism and thus help to select appropriate food type. According to a recent study of Ding et al. [27], digestive enzymes of *G. columna* have the highest content of protease enzymes. This study aims to investigate whether temperature and salinity affect the digestive enzymes profile of *G. columna*. By obtaining the maximum digestive enzymes profile and body composition, the optimal feeding time could be determined and explored to achieve better growth in coral aquaculture.

So far, there are no studies that have reported the effect of temperature and salinity on the growth, physiology, body composition, digestive enzymes activity, oxygen consumption and ammonia nitrogen metabolism of *G. columna*. This study was divided into three experiments to research the above objectives: Experiment 1: Effects of temperature and salinity on *G. columna* adaptability, metabolism and growth; Experiment 2: Effects of temperature and salinity on digestive enzyme activities of *G. columna*. Experiment 3: The diurnal periodicity of digestive enzymes and the optimal feeding time during commercial bait was applied to *G. columna* aquaculture. In addition to discovering the effects of temperature and salinity on the digestive enzymes and physiological metabolism of *G. columna*, this study can also be applied to the large-scale coral aquaculture to promote the sustainable development of coral reefs.

## 2. Materials and Methods

This experiment used 100 *G. columna* colonies obtained from the Taiwan Coral King coral farm (Kaohsiung, Taiwan). These 100 colonies are segmented from the same *G. columna* colonies. Coral farms raise corals with a salinity of 30 ± 0.5 psu and a temperature of 25 ± 0.5 °C. The specimens were maintained and nourished in an aquarium (60 × 35 × 30 cm) with a recirculating filtered seawater system. HME Block 2 Series LED Purple Lights (400–430 nm) were selected as the light source for this study. The LED light frame was set above the glass tank and 30 cm above the water surface. Moreover, a water pump was installed to remove the mucus on the coral’s surface, thereby preventing tissue necrosis. After 2 months of acclimation and self-repair, the healthy corals were segmented into a colony containing 5 polyps and then fixed on porous foundation stones with coral glue. Each treatment group contained 10 colonies. All experiments were carried out in triplicates, and each group was involved with 30 colonies (*n* = 10 colonies). After approximately 72 h of tissue repair, the polyps were examined at full extension, and the experiment was commenced. Water quality was monitored and controlled within a safe range every day [26], which is summarized in Table 1.

### 2.1. Experiment 1: Effects of Temperature and Salinity on G. columna Adaptability, Metabolism and Growth

#### 2.1.1. Temperature and Salinity Control

In this experiment, seawater and RO water were mixed accordingly to obtain different salinity ranges (0–40 psu). The 2000 mL glass beakers of each treatment group were first filled with 1000 mL natural seawater with a salinity of 30 psu as the control group. In the 35 and 40 psu groups, highly saline seawater was further added to increase the salinity, while into the 25 and 20 psu groups was added RO pure water to reduce the salinity; they were measured with the HAND REFRACTOMETER (ATAGO Co. Ltd., Tokyo, Japan). The 2000 mL beakers filled with various salinity waters placed in the fish tanks were constantly maintained at temperatures of 20, 25 and 30 °C. These test beakers were covered with plastic wrap as a barrier to avoid the salinity change caused by the evaporation of water, and the salinity measurement was carried out every week. Aeration was provided in each beaker to maintain important growth parameters such as dissolved oxygen level and homogenous distribution of temperature, pH, nutrient, etc.

#### 2.1.2. Coral Adaptability Test against Changing Temperature and Salinity (Stretching and Contractile Behaviors)

This experiment was carried out to determine the best temperature and salinity range for the aquaculture of *G. columna*, for which the experimental condition was adopted from [21,22]. *Goniopora columna* was captive in each seawater-filled 2000 mL beaker, and the beaker was placed into the aquarium (45 × 27 × 30 cm) with individual temperature control system to maintain 20, 25 and 30 °C temperature independently. A pump provided the required dissolved oxygen. The salinity in the beaker was adjusted to 25, 30, 35 and 40 psu, and the experiment was conducted in triplicates (*n* = 3). The corals were added in a beaker in sequence and each of them was observed for 30 min for their expansion under different temperatures and salinity. It was observed in coral farms that polyps can expand 100% within 30 min under suitable environment. In this study, 30 min was decided (based on reports) to observe percentage expansion. It was observed that once the polyps is fully expanded, it remains expanded until the lights are turned off. The contraction and expansion behavior of the coral’s polyps is scored from 0 to 4, in which 0 was set for complete contraction; 1 was the expansion of the corals (25%); 2 denotes part of the corals fully extended, but not more than half (50%); 3 denotes part of the corals fully extended, more than half (75%); and 4 denotes 100% of the coral’s expansion.

#### 2.1.3. Oxygen Consumption and Ammonia Excretion Test

The seawater was kept in the BOD bottle with precise salinity, and the *G. columna* was inoculated in it. Each experimental group adopted a 3 × 4 complex factor for every machine design, four kinds of salinity (25, 30, 35 and 40 psu); three kinds of water temperature (20, 25 and 30 °C), with a salinity of 30 psu and water temperature of 25 °C as the control group (C). All the sets were carried out in triplicates. The corals were observed under a changing environment of oxygen and ammonia. The changes in dissolved oxygen were determined every hour in the water (YSI 52 DISSOLVED OXYGEN METER, Yellow Springs, OH, USA). In addition, samples were collected from each set to analyze the ammonia concentration in the test water and calculated every 6 h the average ammonia excretion rate. The concentration of ammonia was determined in water according to the method described by Solorzano [28]. Accordingly, 5 mL of test water was mixed with 0.2 mL of phenol-alcohol (10% sol.), 0.2 mL of sodium nitroprusside solution and 0.5 mL of oxidation reaction solution. The color was measured with a spectrophotometer at OD 640 nm, and the ammonia concentration was determined from the standard curve. The average oxygen consumption rate and ammonia excretion rate were determined to obtain the O/N ratio in order to understand the energy metabolism index of the *G. columna*.

#### 2.1.4. Determination of Coral Growth and Polyp Count

Coral growth was determined by total weight and polyp count as described by [29,30]. The tissue and skeleton dry weight were also measured. The algae were removed using brush from the foundation stone prior to taking coral weight. They were then placed on a plastic Petri dish, and the coral weight was measured using an electronic balance after the balance was reset to zero. *G. columna* has a large polyp that can be observed directly with the naked eye. Calculations were made once a week and photographs to record the increased number of new polyps were taken using a Canon EOS 750D camera. The experiment was conducted for 8 weeks, and the growth rate of corals was evaluated during prolonged cultivation. The amount of tissue and skeleton dry weight and polyp was measured every 7 days. Accurate coral weighing was carried out using the drip-dry weights method described by [27,29]. The initial weight of each colony was 1.93 ± 0.53 g. Specific growth rate (SGR), mean value and standard deviation were calculated after the experiment. The SGR of the coral was measured using the following formula:

$$\mathrm{SGR}\;(\%\;{\mathrm{Day}}^{-1})=\left(\frac{In\left(wf\right)-In\left(wi\right)}{\Delta t}\right)\;\times \;100$$

where *wf* is the final weight of the coral (g), *wi* is the initial weight of the coral (g) and Δt is the days of the experimental time.

*Goniopora columna* death was determined based on polyps tissue as the basis for assuming death. After the experiment, a dissecting microscope (Olympus SZX12, Tokyo, Japan) was used to observe tissue residues in the corallites and septa. If no coral tissue was found, the *G. columna* was assumed as dead. The mean and standard deviation (SD) of the survival rate has been calculated. The survival rate of the coral was calculated using the following formula:

Survival rate (%) = (numbers of final alive/numbers of initial samples) × 100

### 2.2. Experiment 2: Effects of Temperature and Salinity on Digestive Enzyme Activities of G. columna

#### 2.2.1. Analysis of Coral Body Composition and Feed

This experiment aimed to examine whether changes in temperature and salinity affect the digestive enzymes and body composition of the *G. columna*. For this, *G. columna* were sonicated and protein concentration was measured by a Bradford protein assay kit (Ameresco, Fountain Parkway, Solon, OH, USA) with Bovine serum albumin as a protein standard. Fat content analysis was done according to the official methods [13,31], lipids were extracted from *G. columna* by hexane; then, subsamples were transferred to test tubes and evaporated to dryness. Total lipids weight was determined and the derived weight values were converted to micrograms. The calculation formula is expressed as follows:

$$\mathrm{Lipid}=\frac{Wi-Wo}{S}\times 100$$

where *Wo* = constant weight of the aluminum cup (g), *Wi* = extracted oil contained in the aluminum cup weight (g) and S = sample weight (g). Carbohydrates were measured based on [32,33], with glucose serving as reference material. Absorption value of 505–660 nm was used to determine glucose content. The formula for glucose content derivation is expressed as follows:

$$\mathrm{Glucose}\;\left(\text{µ}g/\mathrm{mL}\right)=\frac{\mathrm{Sample}\;\mathrm{tube}\;\mathrm{absorbance}}{\mathrm{Standard}\;\mathrm{tube}\;\mathrm{absorbance}}\times \mathrm{Glucose}\;\mathrm{standard}\;\mathrm{concentration}\;\left(µg/\mathrm{mL}\right)$$

Glucose (µg/mL) = [A (sample with colorimetric test − sample)/A (standard tube)] × Glucose standard concentration (µg/mL)

At the end of the experiment, means and standard deviations were calculated.

#### 2.2.2. Analysis of Digestive Enzymes

Enzyme extraction was implemented according to the method of [34]. Protease and lipase extraction were performed with 10 mM sodium citrate buffer (pH 7.0) in a cold environment. Each coral was first rinsed in the buffer solution while adding 10 times volume of buffer solution. The grinding of tissue was performed under ice; thereafter, it was centrifuged at 10,000× *g* at 4 °C for 10 min, and the supernatant was collected and stored at −20 °C. Protease content analysis was carried out as per [34]. Accordingly: 1 mL casein was added in 0.5 mL of enzyme extract and incubated for 15 min, then 1.5 mL of 10% trichloroacetic acid was added. After centrifugation at 6000× *g* at 4 °C for 10 min, the supernatant was collected and for reaction, it was mixed with 5 mL of 0.55 M Na_2_CO_3_ and 1 mL of Folin phenol-staining reagent. The absorbance was measured at 680 nm.

Lipase content analysis was performed according to [35]. One point five mL of olive oil was added in 1.5 mL Tris–HCl (0.1 M buffer, pH 8.0) in which 1 mL of enzyme extract was added before incubation at 37 °C under shaking. After 6 h, 95% alcohol was added in it to terminate the reaction, and then the sample titrated with 0.01 N NaOH until the solution turned brown in color. For this, thymolphthalein containing 0.9% alcohol was used as an indicator.

Amylase content analysis followed as per the method of [36] with slight modification. Accordingly: 0.05 M phosphate buffer solution (pH 7.0) was added in 2% (*w*/*v*) starch solution, and the mixture incubated at 25 °C for 5 min. Then, 1.0 mL of crude enzyme extract was added in 1.0 mL substrate solution and the mixture reacted at 50 °C for 10 min; then, 2 mL of dinitrosalicylic acid reagent (DNS) was added to stop the reaction in a boiling water bath, subsequently cooled, and then the absorbance measured at 520 nm. The maltose was used as standards in DNS assay. Amylase activity was determined as a mole of maltose content converted per minute by per milligram protein. At the end of the experiment, means and standard deviations were calculated.

### 2.3. Experiment 3: Diurnal Changes Analysis of G. columna Body Composition and Digestive Enzymes

The best coral bait (commercial protein-based food) or R1 coral food was obtained from Taiwan Coral King Aquarium company (Pingtung, Taiwan) and was used as control feed in this experiment. *G. columna* was fed in an aquarium (60 × 35 × 30 cm) using a recirculating filtered seawater system. The light and dark cycle times (h) were set for 6 L:18 D. Each treatment group was performed in triplicate for a total of three aquariums. Sampling and analysis were performed at 6:00, 12:00, 18:00 and 24:00 h, respectively. Ten corals were sampled from each aquarium in three replicates. In order to ensure the accuracy of the experiment, sampling was carried out for 3 days in this experiment. After sampling, refer to the method in Section 2.2.1 and Section 2.2.2 above for analysis of coral body composition and digestive enzymes. The purpose of this study was to determine when feeding corals could achieve better digestion and absorption.

### 2.4. Statistical Analysis

The data obtained in these experiments were measured by IBM SPSS statistics 20 (Statistical Package for Social Science) statistical package software, using Two-way ANOVA and Duncan’s Multiple Range Test (DMRT). For the effects of different temperature and salinity on the growth, dry weights of the coral tissue and skeleton, number of polyps, oxygen consumption, ammonia excretion, digestion, metabolism and adaptability of *G. columna*, the significance level was set as *p* < 0.05. Each experiment in this study was carried out in triplicates, denoted as (*n* = 3). In addition, one-way ANOVA and Duncan’s Multiple Range Test (DMRT) were performed on body composition and digestive enzymes detected by Diurnal changes analysis. Before performing the one-way ANOVA analysis, the power analysis was performed first; the power analysis is 0.9, which is in line with the range value. The IBM SPSS statistics 20 (Statistical Package for Social Science) statistical package software was used.

## 3. Results

### 3.1. Experiment 1: Adaptation, Metabolism and Growth of G. columna

#### 3.1.1. Growth and Survival

**Figure 1:**
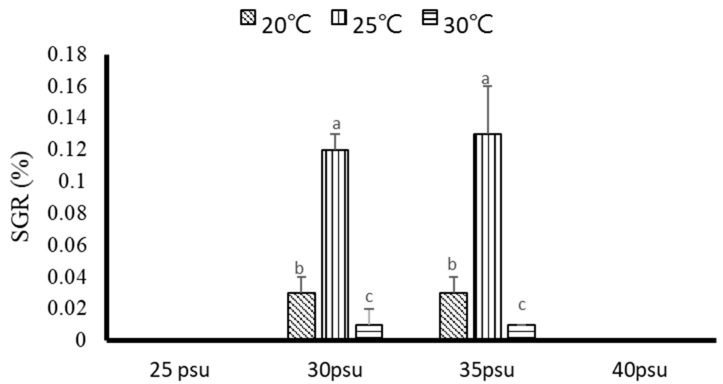
The growth performance of *G. columna* after 8 weeks of rearing at different temperatures and salinities. Each Value represents ±SD (*n* = 3). The Latin alphabet represents significant differences (*p* < 0.05) in the case of different letters and no difference when the same letter appears.

In this experiment, the growth and survival of corals were investigated for four salinity and three temperature ranges, and the growth was determined based on the number of polyps increased. As shown in Table 2, after 8 weeks, it showed a better growth at 30 to 35 psu and temperature 25 °C. For the % SGR, as shown in Figure 1, 0.13 ± 0.03% obtained at salinity of 35 psu and 0.12 ± 0.01% at salinity of 30 psu and temperature 25 °C were the best SGR, with significant differences compared with other groups (*p* < 0.05). Salinity of 35 psu with a temperature of 30 °C and salinity of 40 psu with all temperature conditions have shown the lowest number of polyps. This study clearly showed that when the salinity of the *G. columna* aquaculture was adjusted to salinity 30 and 35 psu and temperature 25 °C, the polyp numbers were maximum, which was determined as the most suitable parameter range for the maximum growth of *G. columna*.

**Figure 2:**
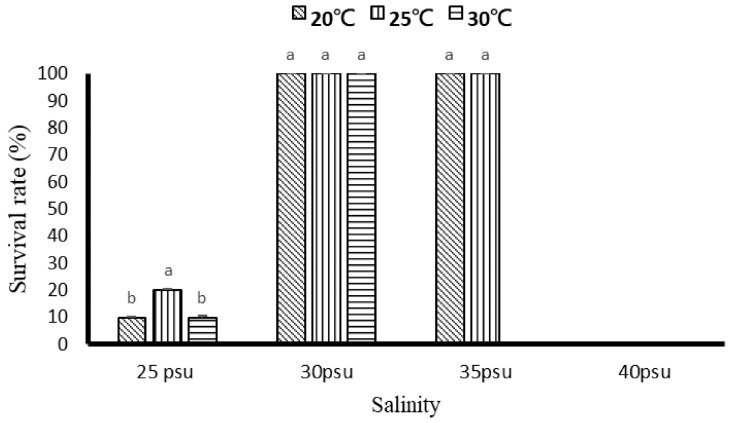
The effect of 8 weeks of different temperature and salinity on the survival of *G. columna*. Each value represents ± SD (*n* = 3). The Latin alphabet represents significant differences (*p* < 0.05) in the case of different letters and no difference when the same letter appears.

The results of the survival rate are shown in Figure 2. The survival rate was 100% in the entire temperature range tested under 30 psu salinity group, and with 20 and 25 °C under the 35 psu salinity group. However, when the temperature was higher than 30 °C, under the 35 psu salinity group set, the survival rate was greatly affected and obtained equal to 0%. In the salinity group 25 psu, all the temperature ranges have shown the survival rate of approx. 10–20%. The results show that when the salinity was higher up to 40 psu, the coral polyps stopped their growth in all temperature ranges, and in the majority of cases, they undergo polyps atrophy or death.

#### 3.1.2. The Effect of Temperature and Salinity on Coral OXYGEN Consumption

Under different temperature and salinity ranges, the oxygen consumption rate of corals was significantly changed. At 30, 35 psu salinity and temperature of 25 °C, the oxygen consumption rate was higher than the other groups (Table 3). The oxygen consumption rate under different salinity conditions and three temperatures (20, 25 and 30 °C) were determined. From the salinity of 25 psu, the oxygen consumption rate was, respectively, 0.10 ± 0.01, 0.20 ± 0.01 and 0.30 ± 0.02 mg/L; from 30 psu salinity, oxygen consumption was 2.50 ± 0.02, 2.40 ± 0.05 and 2.10 ± 0.07; whereas 2.10 ± 0.05, 2.60 ± 0.03 and 0.10 ± 0.01 mg/L oxygen consumption rate was recorded at 35 psu. From salinity 40 psu, oxygen consumption was at least 0.10 ± 0.01 mg/L with all three temperatures (20, 25 and 30 °C). In conclusion, the oxygen consumption rate was the highest at 35 psu salinity and 25 °C temperature, increased by 1 to 25 times compared with other groups, which was significantly different from other groups (*p* < 0.05).

#### 3.1.3. The Effect of Temperature and Salinity on Ammonia Metabolism

*G. columna* has shown the variability in discharge of ammonia under different temperature (20, 25, and 30 °C), and salinity (25, 30, 35 and 40 psu) conditions; however, at 30 and 35 psu salinity with all temperature ranges, discharge of ammonia was the highest recorded (Table 3). None of the other groups have shown a significant difference in ammonia discharge. Table 3 shows that under the conditions of 20, 25 and 30 °C, the ammonia nitrogen content at 25 psu salinity was 0.02 ± 0.01, 0.03 ± 0.01 and 0.02 ± 0.01; from the salinity of 30 psu, nitrogen was 0.14 ± 0.02, 0.13 ± 0.04 and 0.12 ± 0.03 mg/L; from the salinity of 35 psu, 0.14 ± 0.02, 0.16 ± 0.05 and 0.01 ± 0.01 mg/L; and from the salinity of 40 psu, nitrogen was constant, 0.01 ± 0.01 mg/L. The results showed that the ammonia excretion rate was the highest when the salinity and temperature was 35 psu and 25 °C, which was significantly different from the other groups (*p* < 0.05).

#### 3.1.4. Energy Metabolism Rate

Using ammonia nitrogen and the oxygen consumption rate as the energy metabolism index, the O/N ratio was calculated in *G. columna*. In Table 2, with the temperature 20, 25 and 30 °C, the 25 psu salinity metabolic rates obtained, respectively, 5.00 ± 1.25, 6.67 ± 1.09 and 15.00 ± 1.32; from the 30 psu salinity, 17.86 ± 1.43, 18.46 ± 1.45 and 17.50 ± 1.39 metabolic rates were recorded; when the salinity was 35 psu, the metabolic rates were 15.00 ± 1.29, 16.25 ± 1.24 and 10.00 ± 1.58; and from salinity 40 psu, the metabolic rate was constant at 10.00 ± 0.10. The results show that all groups with values < 24 belong to the protein oxidation type metabolism, and obtained a higher ratio at a salinity of 30 psu and temperature of 25 °C. The ratio was the lowest at a low temperature 20 °C and a low salinity of 25 psu. The results showed that when salinity and temperature changes, it will affect the energy metabolism of *G. columna*. Based on this outcome, regarding the choice of bait, protein-based feed can be selected to enhance the growth of corals.

#### 3.1.5. The Effect of Temperature and Salinity on *G. columna* Adaptability

**Figure 3:**
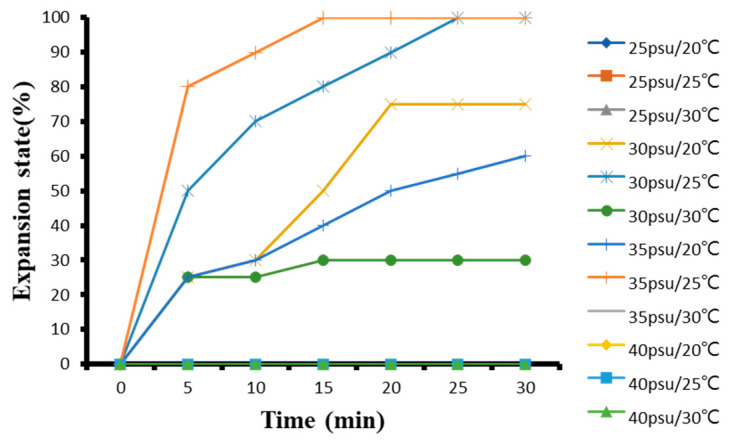
The influence of different temperature and salinity environment on the adaptability of *G. columna*.

In response to different temperature and salinity stress, the polyp expansion rate at 35 psu and 25 °C was up to 80%, at 30 psu and 25 °C, polyps could expand up to 50%, and 25 and 40 psu groups both have exhibited 0% expansion (Figure 3). The results of the 30-min test showed that the *G. columna* in the group with salinity of 30 and 35 psu at 25 °C can reach 100% expansion, the salinity of 30 psu and temperature 20 °C expansion was 75%, the salinity of 35 psu at 20 °C expansion was 60%, the salinity 30 psu with temperature of 30 °C expansion was 30%, and the other groups were exhibiting 0% polyp expansion (Figure 3). The results of the coral temperature and salinity adaptability test show that a salinity of ~30–35 psu and temperature of 25 °C provided the most suitable condition for the expansion of the *G. columna* tentacles. Table 3 illustrates the score of polyps stretching and contractile behaviors.

### 3.2. Experiment 2: Changes in Digestive Enzyme Activity

#### 3.2.1. The Influence of Temperature and Salinity on *G. columna* Body Composition

**Figure 4:**
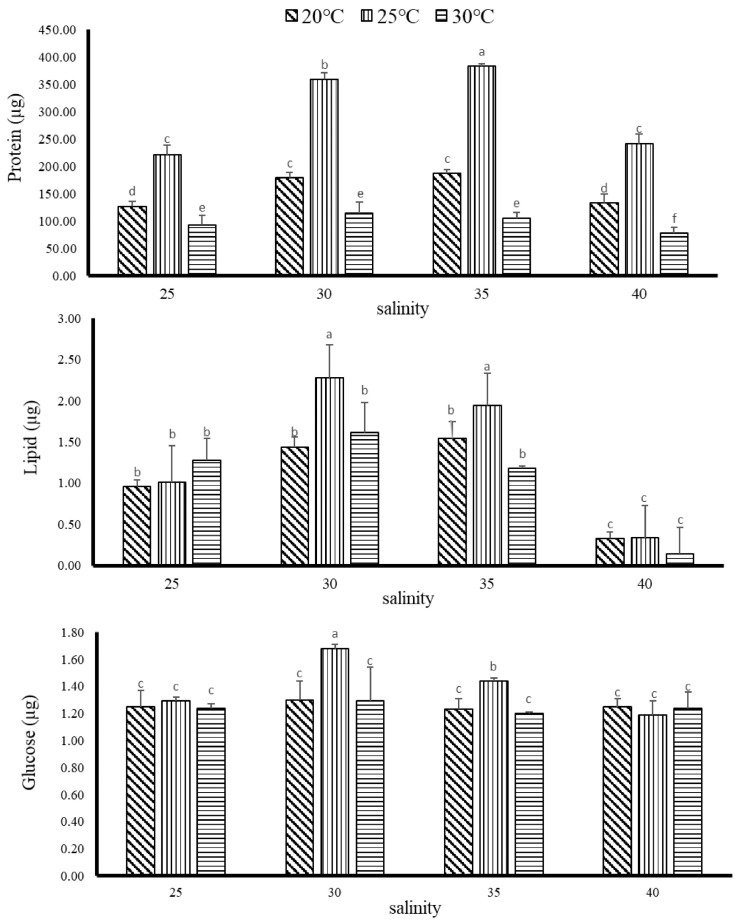
Effects of 8 weeks of rearing at different temperatures and salinities on the composition of *G. columna*. Each value represents ±SD (*n* = 3). The Latin alphabet represents significant differences (*p* < 0.05).

After 8 weeks of temperature and salinity test, it was found that the digestive enzymes and body composition of *G. columna* were changed. The results are shown in Figure 4. The protein content was highest at the salinity of 35 psu and a temperature of 25 °C, which was significantly different from other groups (*p* < 0.05). The salinity of 30 psu and the temperature of 25 °C were the second best, and they also showed significant differences with the other groups (*p* < 0.05). In normal seawater with the salinity of 30 psu and temperature of 30 °C, the protein content was 3.1% lower than that at 25 °C. When the salinity was higher than 40 psu and a temperature of 25 °C, the protein content was significantly lower than that obtained at 35 psu and 25 °C, which was also showing a significant difference (*p* < 0.05). In addition, the protein content of the two groups of 20 °C and 30 °C under the salinity of 25 psu was lower than that of the 25 °C group. The results show that the level of temperature and salinity significantly affect the protein content of *G. columna*. Likewise, *G. columna* body fat has a higher content (1.94–2.28) from the salinity of 30 and 35 psu at a temperature of 25 °C (*p* < 0.05), and the maximum glucose fraction (1.44–1.68) from a salinity of 30 and 35 psu with a temperature of 25 °C than that of other groups (*p* < 0.05).

#### 3.2.2. The Effect of Temperature and Salinity on Digestive Enzyme Activity of *G. columna*

**Figure 5:**
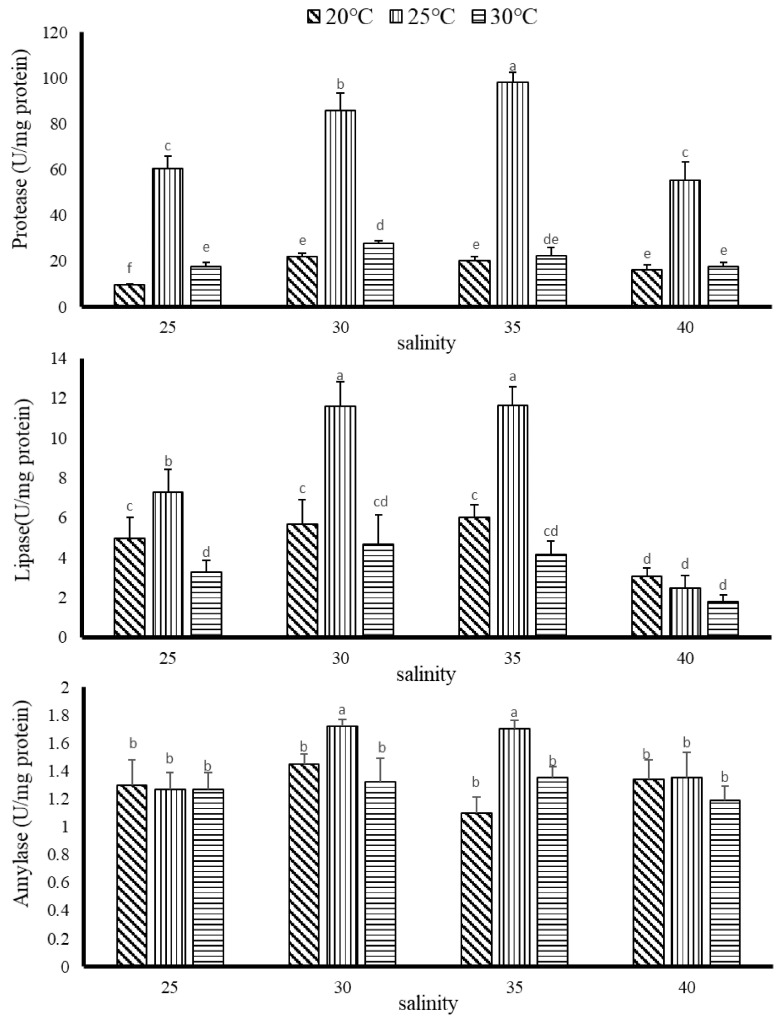
The activity of digestive enzymes in *G. columna* coral after 8 weeks of feeding at different temperatures and salinities. Each value represents ±SD (*n* = 3). The Latin alphabet represents significant differences *(p* < 0.05).

The effects of temperature and salinity on the activity of coral digestive enzymes are shown in Figure 5. The comparative account showed that the protease activity was highest at 35 psu salinity with a temperature of 25 °C, which was significantly different from other groups (*p* < 0.05). The salinity of 35 psu and the temperature of 25 °C increased by an average of 2.94 times compared with the other treatment groups; the major difference was 40 psu and 20 °C, with a difference of 6.03 times. The results from the 30 psu salinity group with the temperature 25 °C were significantly different from the other experimental groups (*p* < 0.05). A decrease in protease activity by 3.1 and 3.9% was recorded from the 30 psu salinity group with the temperature of 30 and 20 °C as compared to 25 °C, whereas the protease activity was lower when the salinity was 40 psu and 25 °C. There was a significant low protease activity when the salinity was other than 35 psu at 25 °C (*p* < 0.05). In addition, the protease activity of the other two temperatures of 20 °C and 30 °C were always lower than 25 °C under all salinity ranges tested. However, their values were apparently increased with increasing salinity up to 35 psu. The results clearly show that temperature and salinity both have a great effect on protease activity in corals.

The comparison of lipase activities among the three different salinities at 20, 25 and 30 °C showed that the highest activity was at 30 and 35 psu and 25 °C, which was 11.62 ± 1.20 U/mg and 11.65 ± 0.94 U/mg; there was a significant difference compared with other groups (*p* < 0.05). When the salinity and the temperature were 40 psu and 30 °C, the lipase activity was the lowest at 1.78 ± 0.37 U/mg, which was 6.53 and 6.54 times lower than the two groups with the best activity, respectively.

The analysis of amylase activity in *G. columna* showed, in moderate range, all values were lower than 1.72 U/mg protein. At salinity 30, 35 psu and temperature 25 °C, the best amylase activity was 1.72 ± 0.05 and 1.70 ± 0.06 U/mg, which were significantly different from other treatment groups (*p* < 0.05).

### 3.3. Experiment 3: Diurnal Variation of Digestive Enzyme Activity and Body Composition

#### Diurnal Changes Affecting Coral Body Composition and Digestive Enzymes Profile

**Figure 6:**
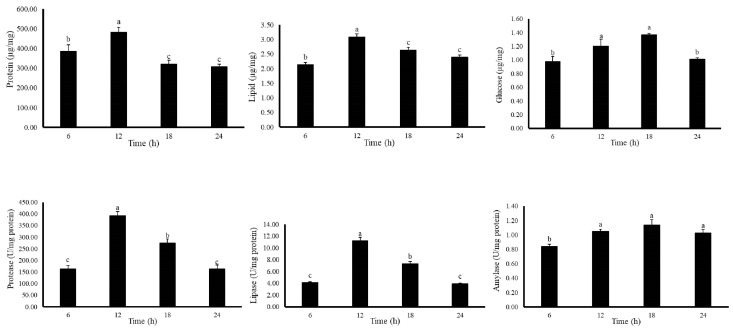
Twenty-four hour changes in activity of body composition and digestive enzymes of *G. columna* in commercial R1 coral food. Each value represents ±SD (*n* = 3). The Latin alphabet represents significant differences (*p* < 0.05).

The changes of body composition and digestive enzymes of *G. columna* were observed by feeding coral food R1 throughout the 24 h period. As shown in Figure 6, the protein content in *G. columna* at 6:00; 12:00; 18:00 and 24:00 h was 358.39 ± 32.19, 482.34 ± 25.21, 321.12 ± 19.28 and 307.32 ± 13.29 μg, respectively. The protein content at 12:00 was 1.35, 1.50 and 1.57 folds higher than 6:00, 18:00, 24:00 h samples, respectively (F(3,8) = 2739.072, *p* < 0.001). Lipid was low in *G. columna* and ranging between 2.14–3.09 µg (F(3,8) = 9.258, *p* < 0.006). The glucose content of each group was about 0.98–1.37 µg, the content was higher at 12:00 and 18:00 h, and there was a significant difference compared with the other two times (F(3,8) = 230.871, *p* < 0.001).

As shown in Figure 6, the protease activity in 6:00, 12:00, 18:00 and 24:00 h was 163.21 ± 15.11, 391.35 ± 19.16, 257.31 ± 16.93 and 162.34 ± 17.21 U/mg protein. The protease activity at 12:00 h was 2.40, 1.52 and 2.41 folds higher than 6:00, 18:00, 24:00 h, respectively (F(3,8) = 2978.197, *p* < 0.001). There was no transitional difference in the lipase (F(3,8) = 102.003, *p* < 0.001) and amylase (F(3,8) = 6.076, *p* < 0.019) activity contents between the groups. The activity of digestive enzymes was compared with the protein content in vivo. The protein content was lower when the digestive enzyme was low. At 12:00 h, the protein content was the highest, and the digestive enzyme activity was also the highest. In the evening, the activity of digestive enzymes decreased, the content of body composition protein was also decreased, and at the lowest activity of 00:00 h, body composition protein and digestive enzyme were also the lowest. Therefore, it can be inferred from the experimental results, the digestion and absorption of *G. columna* will have a daily cyclic change. The most suitable feeding time for coral culture was from 6 a.m. to 12 noon, when the activity of digestive enzymes gradually increases, and the digestion of feed and higher nutrient absorption could have maximum benefit towards growth.

## 4. Discussion

### 4.1. Growth and Survival

Corals are lower animals with extremely strict requirements for temperature and salinity. Results showed a significant increase in *G. columna* growth at 30–35 psu salinity and 25 °C temperature. When the temperature of seawater rises to 30–32 °C, coral viability is affected, and beyond that its death was observed. This corresponds with the finding by Glynn and D’croz [8] that the stony coral *Pocillopora damicornis* grows optimally between 26–28 °C. Therefore, the results of this experiment clearly showed that in two salinity ranges of 25 or 40 psu, there was always a lower coral growth and survival rate recorded. When the temperature was 20 °C or 30 °C, the growth would stagnate and chlorophyll loss occurred; however, it did not cause the coral death. It shows a greater impact on their lives with salinity change; it is even leading to loss of chlorophyll or coral death [18]. Changes in osmotic pressure also stimulate corals to excrete symbiotic algae from their bodies to reduce their metabolic rate, resulting in chlorophyll loss in coral, and their respiration rate also is reduced to retain energy and to resist environmental changes. A study of nine different corals, *Galaxea fascicularis*, *Montipora capricornis*, *Montipora capricornis*, *Turbinaria reniformis*, *Echinopora lamellose*, *Acropora tenuis*, *Pocillopora damicornis*, *Stylophora pistillata* and *Psammocora contigua*, found that they had a stress response when exposed to 30 °C and 20 psu salinity. It can increase the activities of superoxide dismutase (SOD), Catalase (CAT) and Glutathione stransferase (GST) in these corals, and most of them died after 60 days due to low salinity environment, whereas *G. fascicularis* and *P. contigua* died because of high temperatures [13]. In addition, genetic adaptation between corals and Symbiodinium was also found to adapt the extreme environmental changes. However, once the environmental changes go beyond their tolerance limit, it can cause death [37,38]. The optimal aquaculture conditions of *G. columna* are 30–35 psu and 25 °C. Therefore, climate change will cause dramatic changes in seawater temperature and salinity, which will cause *G. columna* bleaching or death. This study preliminarily found that the optimal breeding conditions for *G. columna* are 30–35 psu and 25 °C. If the temperature and salinity changes caused by climate change exceed the tolerable range of G coral, in addition to causing bleaching, it will also affect body composition and digestive enzymes, which in turn affects physiological metabolism and oxygen consumption. Previous studies have found that salinity below 26 psu will slow the growth of *Platygyra acuta*, and a drop to 22 psu will cause developmental stagnation [6]. In addition, it will also cause the eggs of *Acropora millepora* to not be fertilized smoothly [5]. Therefore, the greater the changes in temperature and salinity caused by environmental changes, the more severe the physiological and metabolic threats to corals.

### 4.2. Body Composition and the Influence of Digestive Enzymes

This study initially found that the body composition and digestive enzymes of *G. columna* are affected by changes in temperature and salinity environment. The digestive enzymes of *G. columna* have been addressed for higher protease content by a previous study [27]. When the salinity was 30–35 psu and 25 °C, the protein content in the *G. columna* body was higher than that of the other groups. In addition to the significant difference in the protein content in *G. columna*, the protease content was also found to have relatively higher enzyme activity under the conditions of 30–35 psu and the 25 °C salinity-temperature group; whereas at high temperature with high or low salinity, the protease activity in the *G. columna* body usually is lower. Moreover, the glucose and lipid content were very low, and the amylase and lipase activities were also very small. This result and the O/N ratio result both show that *G. columna* mainly uses protein as one of the main energy sources in nutrient utilization. When the temperature is low, the metabolic rate decreases, which causes energy to be used to maintain body temperature, which in turn affects the efficiency of food digestion and absorption. On the other hand, when the temperature rises, the metabolism speeds up [12,14,15,39]. This experiment confirmed that climate change will affect the digestion and metabolism of corals. Harriott (2002) found that temperature and salinity can affect the physiochemical changes of corals, thereby causing coral stress response, which has a great impact on global coral reef proliferation and coral species diversity [40]. Therefore, the drastic changes in temperature and salinity caused by environmental changes pose a threat to the sustainability of coral reefs. In order to promote the sustainable development of coral reefs, it is an issue worthy of attention to control the temperature and salinity within the range suitable for coral survival and to conduct large-scale coral aquaculture.

Under normal circumstances, the body temperature of temperature-changing organisms was the same as the water temperature of the living environment [41]. Aquatic organisms are affected by temperature, and they may behave differently in ways or degrees, but there will basically be a sudden increase in metabolism, and then a process of gradually stabilizing [42]. Living organisms at low temperatures can cause hypoxia in the biological central nervous system and insufficient energy release, causing the metabolic rate to be unable to maintain individual life, causing changes in the ion balance in cells and causing death [43]. Changes in salinity will cause marine organisms to face physiological adjustment problems such as ion diffusion and osmotic pressure. Mantel and Farmer [44] pointed out that the ability to adapt to osmotic pressure in salinity can be divided into euryhaline and stenohaline. Corals are of narrow salinity and have poor ability to regulate osmotic pressure. If organisms cannot effectively and quickly adjust osmotic pressure, they will die within a short period of time [45]. Therefore, when the temperature and salinity change, it will have a great physiological impact on the feeding and digestion of corals.

### 4.3. Physiological Metabolism

The results of this study showed that the highest O/N and average ratio are, respectively, 19.92 ± 1.45 and 12.8 ± 1.03, which are less than 24. It belongs to the protein oxidation type and mainly uses protein as an energy source [26]. Therefore, a protein diet must be selected for the best growth promotion of corals. The results show that when the salinity is higher than 40 psu, the O/N ratio at 20–30 °C was close to 10.00 ± 0.10; when the salinity was 25 psu, O/N ratio was, respectively, 15.00 ± 1.32 and 5.00 ± 1.25 at the 30 psu salinity and 20 °C temperature; noticeably, the O/N difference between both temperatures was threefold, which clearly shows that temperature and salinity certainly affect the energy metabolism rate of *G. columna*. Studies have confirmed that after a few hours of salinity drop from 30 psu to 10 psu, there was a negative effect on the metabolism and photosynthesis efficiency of two corals, *Porites lutea* and *Pocillopora damicornis* [46]. Therefore, the greatest impact of salinity changes on corals results in changes in their physiological metabolism and respiration rate. According to a previous study, reduced salinity significantly reduced the process of appropriate larval settlements of *Acropora hyacinthus*, *Favites abdita*, *Platygyra daedalea* and *Platygyra sinensis* [47]. *Platygyra acuta*, however, had no significant effect on its Larval settlement due to unfavorable temperature and salinity changes. Therefore, larvae of different coral species have different tolerance to warm salinity [48]. Therefore, the greatest impact of salinity changes appeared on corals in the form of their physiological metabolism and respiration rate variations. In recent years, due to climate change, ocean temperature and salinity have changed drastically. Higher temperature will lead to excessive evaporation of water, which will also increase the salinity of seawater. These two factors will greatly affect the energy metabolism of corals.

### 4.4. The Effect of G. columna Adaptability and Best Time for Feeding

This study preliminarily found that changes in temperature and salinity caused by climate change would threaten the physiological metabolism and digestive enzymes of *G. columna*. Therefore, we further explored whether changes in temperature and salinity would affect changes in polyps feeding and body composition. After a 30-min feeding test, the *G. columna* exposed to the salinity 30–35 psu and temperature 25 °C group could exhibit 100% expansion. Preliminary studies have found, in the salinity range 30–35 psu with the temperature 25 °C, *G. columna* could enhance the growth, survival and adaptability, which can be set as the most suitable temperature and salinity range for their breeding. Unsuitable temperature and salinity conditions will cause coral polyps to shrink, which in turn affects feeding and body composition. This was also the case in previous studies; when the salinity of seawater drops to 15 psu in an instant, it is found that the polyps of *Pocillopora damicornis* and *Montipora verrucosa* shrink and in the course of time, gradually they die [49]

The effect of feed timing studies on *G. columna* body composition reveals that the protein content in the body was maximum 482.34 ± 25.21 µg at 12:00 h, and significantly decreased (ranging from 321.12–358.39 µg) in the remaining hours (18:00, 00:00 and 6:00 h (the next day)). It was found that the protein content in *G. columna* increased to the highest level within 6 h after feeding, and the digestive enzyme decreased after 6 h. The digestion time was determined to be about 6 h. According to Sebens and Koehl [50], the digestion time of different corals is different. The digestion time of *Alcyonium siderium* fed Zooplankton is about 4–6 h. *G. fascicularis* begins to digest within 10 min after eating and finishes in 180 min [51]. Therefore, the digestion and absorption of corals will change periodically from day to day [52,53]. According to the results of the experiment, the most suitable feeding time for corals is between 6:00 and 12:00, when the activity of digestive enzymes was gradually enhanced and the absorption of the bait can exert the maximum benefit. Therefore, to promote coral reef sustainability through large-scale coral aquaculture, controlling the environment at 25 °C, 30–35 psu, and providing feeding will promote the digestion, metabolism and growth of *G. columna*. In addition, if the temperature and salinity exceed the above range, it may affect the physiological metabolism and digestion of *G. columna*, which will threaten the survival of *G. columna*. Previous studies have shown that changes in ambient temperature, which can also lead to genetic variation between corals and zooxanthellae, can also affect the photosynthetic efficiency of zooxanthellae within corals [9,37,38]. Therefore, the impact of salinity variability induces changes in coral’s physiological metabolism and respiration rate. In addition, it removes excess symbiotic algae to reduce its own metabolic rate, and the zooxanthellae, chlorophyll a and respiration rate in order to retain energy to resist temporary interference caused by environmental changes [54]. But long-term exposure to a bad environment would cause coral death. Therefore, corals should not be cultured at unfavorable salinity ranges, so as not to affect the growth and survival of corals. At present, climate change has caused dramatic changes in sea temperature and salinity. Large-scale coral aquaculture in the sea may result in the inability of corals that are sensitive to temperature and salinity to survive. Indonesia is one of the leading countries for coral aquaculture in the sea [55]. Recent studies have pointed out that climate change has caused serious damage to coral reef ecology in Bali, Indonesia [56]. Tito et al. found that between 2015 and 2017, the sea temperature in Bali gradually increased, which also led to coral death in Bali, and the most serious coral death occurred in the northern reef flat [57]. Therefore, as climate change poses a threat to corals, it is necessary to carry out large-scale coral aquaculture indoors to effectively control temperature and salinity, so as to improve the effectiveness of coral cultivation.

## 5. Conclusions

This study preliminarily found that temperature, salinity and feeding time had a great influence on the growth, metabolism and quick adaptability of *G. columna*. Therefore, changes in temperature and salinity caused by climate change do pose a threat to *G. columna* survival. According to the experimental results, the optimal survival conditions of *G. columna* are 25 °C and 30–35 psu. When the temperature and salinity are too high or too low, the physiological metabolism will be affected. The most suitable feeding time is from 6:00 a.m. to 12:00 noon, with high protein and protease activity. This research can not only understand the threat of the coral physiological metabolism caused by temperature and salinity, but also can be applied to large-scale coral aquaculture, which will be very important for the sustainable development of coral reefs. This study has carried out large-scale *G. columna* aquaculture at the Taiwan Coral Kings coral farm. This study preliminarily explores the effects of temperature and salt stress on *G. columna* digestive enzymes and body composition. In the future, more corals or changes in different environmental variables on coral body composition and digestive enzymes can be explored. This information can be used for application in large-scale coral aquaculture or sustainable development of coral reefs.

## Figures and Tables

**Table 1:** The water quality conditions in the study (SD ± 56 day).

Water Quality Conditions	Optimum
pH	8.0 ± 0.31
Ammonia nitrogen	0.04 ± 0.04 mg/L
Nitrous acid	0.02 ± 0.01 mg/L
Nitric acid	0.10 ± 0.01 PPM
Calcium	400 ± 32.03 PPM
Magnesium	1280 ± 63.21 PPM
Phosphate	0.02 ± 0.01 PPM

**Table 2:** Growth, ammonia excretion, oxygen consumption and energy metabolism rate of *G. columna* after 8 weeks of rearing at different temperatures and salinities.

Salinity and Temperature												
	25 psu			30 psu			35 psu			40 psu		
	20 °C	25 °C	30 °C	20 °C	25 °C	30 °C	20 °C	25 °C	30 °C	20 °C	25 °C	30 °C
Initial Polyps (Number ± 95%)	5.00 (0.00)	5.00 (0.00)	5.00 (0.00)	5.00 (0.00)	5.00 (0.00)	5.00 (0.00)	5.00 (0.00)	5.00 (0.00)	5.00 (0.00)	5.00 (0.00)	5.00 (0.00)	5.00 (0.00)
End Polyp (Number ± 95%)	7.67 ^d^ (0.44)	7.67 ^d^ (0.89)	7.33 ^d^ (0.44)	12.33 ^b^ (1.78)	38.33 ^a^ (1.11)	9.33 ^c^ (0.44)	13.67 ^b^ (1.11)	39.33 ^a^ (1.11)	6.00 ^e^ (0.00)	5.00 ^e^ (0.00)	5.00 ^e^ (0.00)	5.00 ^e^ (0.00)
Net Increase (Number ± 95%)	2.67 ^d^ (0.44)	2.67 ^d^ (0.89)	5.33 ^b^ (0.44)	7.33 ^b^ (1.78)	33.33 ^a^ (1.11)	4.33 ^c^ (0.44)	8.67 ^b^ (1.11)	34.33 ^a^ (1.11)	1.00 ^e^ (0.00)	0.00 ^f^ (0.00)	0.00 ^f^ (0.00)	0.00 ^f^ (0.00)
Ammonia nitrogen (mg/L)	0.02 ^c^ (0.01)	0.03 ^c^ (0.01)	0.02 ^c^ (0.01)	0.14 ^ab^ (0.02)	0.13 ^ab^ (0.04)	0.12 ^ab^ (0.03)	0.14 ^ab^ (0.02)	0.16 ^a^ (0.05)	0.01 ^c^ (0.01)	0.01 ^c^ (0.01)	0.01 ^c^ (0.01)	0.01 ^c^ (0.01)
Oxygen consumption (mg/L)	0.10 ^d^ (0.01)	0.20 ^d^ (0.01)	0.30 ^e^ (0.02)	2.50 ^b^ (0.02)	2.40 ^b^ (0.05)	2.10 ^c^ (0.07)	2.10 ^c^ (0.03)	2.60 ^a^ (0.05)	0.10 ^d^ (0.01)	0.10 ^d^ (0.01)	0.10 ^d^ (0.01)	0.10 ^d^ (0.01)
O/N	5.00 ^d^ (1.25)	6.67 ^d^ (1.09)	15.00 ^b^ (1.32)	17.86 ^ab^ (1.43)	18.46 ^a^ (1.45)	17.50 ^ab^ (1.39)	15.00 ^b^ (1.29)	16.25 ^ab^ (1.24)	10.00 ^c^ (1.58)	10.00 ^c^ (0.10)	10.00 ^c^ (0.10)	10.00 ^c^ (0.10)

The superscript Latin alphabet to the right of values indicates horizontal comparisons, and showed significant differences (*p* < 0.05). ± is SD (*n* = 3).

**Table 3:** Score of polyps stretching and contractile behaviors at salinity 30–35 psu and temperature 25 °C.

Score	0	1	2	3	4
Tentacle form	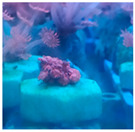	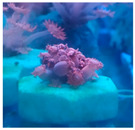	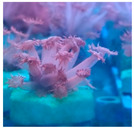	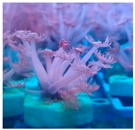	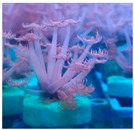
behaviors	Polyps fully contracted	Polyps were slightly elongated	The polyps begin to stretch, but the total length does not exceed 50%.	The polyps begin to stretch, but the total length does not exceed 75%.	Polyps fully extended

## Data Availability

Not applicable.
